# Cardio-respiratory autonomic responses to nociceptive stimuli in patients with disorders of consciousness

**DOI:** 10.1371/journal.pone.0201921

**Published:** 2018-09-12

**Authors:** Guya Devalle, Paolo Castiglioni, Chiara Arienti, Carlo Abbate, Anna Mazzucchi, Luca Agnello, Giampiero Merati

**Affiliations:** 1 Vegetative State Unit, IRCCS Fondazione Don Carlo Gnocchi, Milan, Italy; 2 IRCCS Fondazione Don Carlo Gnocchi, Milan, Italy; 3 Geriatric Unit, Fondazione IRCCS Ca’ Granda, Ospedale Maggiore Policlinico, Milan, Italy; 4 Rete Gravi Cerebrolesioni Acquisite, IRCCS Fondazione Don Carlo Gnocchi, Santa Maria dei Servi, Parma, Italy; 5 Department of Biomedical Sciences for Health, University of Milan, Milan, Italy; 6 Center of Sports Medicine, IRCCS Fondazione Don Carlo Gnocchi, Milan, Italy; University of British Columbia, CANADA

## Abstract

The autonomic response to pain might discriminate among consciousness disorders. Therefore, aim of this study was to describe differences between minimally conscious state (MCS) and unresponsive wakefulness syndrome (UWS) patients in their autonomic response to a nociceptive stimulus. ECG, respiration, finger blood pressure (BP) and total peripheral resistances (TPR) were continuously recorded before, during and after a standardized noxious stimulus in 20 adult brain-injured patients, 14 in UWS and 6 in MCS. Occurrence of fast autonomic responses synchronous with the stimulus was detected by visual inspection of the tracings; short-term (<20 s) and long-term (between 20s and 50 s from the stimulus) responses were evaluated by beat-by-beat quantitative analysis. The noxious stimulus elicited fast responses in both groups, but only MCS patients showed a significant short-term response in TPR and long-term response in HR. Thus, short- and long-term cardiovascular responses to pain might integrate neuro-behavioural assessments to discriminate between MCS and UWS.

## Introduction

In the healthy individual, the cardio-respiratory autonomic response to pain induces an acute and transient increase in heart rate (HR), blood pressure (BP) and ventilation [1]. Such reaction involves a complex neural network, including spinal and trigeminal dorsal horns, brainstem, hypothalamus, amygdale, thalamus and insular cortex [2]. Thus, the reflex autonomic response to a painful stimulus might be altered after a severe brain damage, with severity and localization of the brain damage affecting the autonomic pain reflex.

Therefore, it is conceivable that the evaluation of the autonomic response to pain may give objective information for discriminating states with different alterations of consciousness that follow brain damages, in particular, distinguishing between unresponsive wakefulness syndrome (UWS, i.e. wakefulness without behaviour) and minimally conscious state (MCS, non-reflexive or purposeful behaviour with lack of communication) [3]. Such distinction is clinically important because UWS and MCS patients have different prognoses and a correct diagnosis may support clinicians in taking end-of-life decisions [4]. However, the diagnostic discrimination between these two alterations of consciousness is still puzzling, and the neuro-behavioral evaluations commonly executed in clinical routine requires an expert operator and may lead to misdiagnosis [5].

In this context, response to a painful stimulus might help improving the diagnostic accuracy in discriminating between MCS and UWS. Actually, it has been reported that the areas of the so-called pain matrix are less activated by a noxious stimulus in UWS than in MCS patients, and that MCS patients, unlike UWS ones, preserve the functional connectivity between the primary somatosensory cortex and fronto-parietal associative cortices, suggesting a less impaired capacity of pain perception in MCS patients [6].

Surprisingly, the evaluation of pain response has been judged of minor importance in the clinical assessment of consciousness disorders [7], probably due to the lack of a detailed description of the response to noxious stimuli in MCS and UWS patients. Recently, however, the Nociception Coma Scale (NCS) has been introduced and validated in clinical practice [8]. Its aim is to evaluate the behavioural changes in response to a nociceptive stimulus, and therefore it does not include measures of the autonomic response to pain. Moreover, the NCS evaluation has never been compared to a concomitant cardiovascular autonomic assessment, to understand whether the information provided by behavioural and autonomic responses to pain may be complementary.

Aim of this study is therefore to describe differences between MCS and UWS patients in their autonomic and behavioural reactions to a standardized nociceptive stimulus, in this way evaluating the feasibility of discriminating between states of consciousness from the response to pain. This will be done considering patients classified in UWS or MCS according to the validated Coma Recovery Scale-Revised (CRS-r)[9], and by comparing their response to a nociceptive stimulus in terms of cardiovascular and respiratory autonomic activations and of behavioural response, as assessed by the NCS.

## Material and methods

The study was approved by the ethics committee of Fondazione Don C. Gnocchi, and all clinical investigations have been conducted according to the principles expressed in the Declaration of Helsinki. Due to the clinical condition of the patients, they completely lacked the capacity to provide any consent to the study. Therefore, the informed consent was obtained by the legal surrogate of each enrolled patient.

### Subjects

Twenty patients with severe brain injuries (10 males; age 54±14 years; weight 57.9±7.4 kg; height 167±10 cm, mean±SD) consecutively admitted to our “long-term care unit” (Vegetative State Unit, IRCCS, Don C. Gnocchi Foundation, Milan, Italy) were enrolled. The aetiology was post-anoxic (n = 9), vascular of ischaemic (n = 4) and haemorrhagic (n = 2) origin, traumatic (n = 4), and post-encephalitic (n = 1). Fourteen patients (70%) were currently treated with β-blocking agents (carvedilol, atenolol, bisoprolol). All patients did not need assisted ventilation but one, who was assisted by nocturnal mechanical ventilation only.

Some days before the administration of the nociceptive stimulus, a neuropsychologist executed a behavioural evaluation to each patient, administering the CRS-r [9], to clinically differentiate between MCS and UWS. This validated scale evaluates auditory, visual, verbal and motor functions, communication and arousal, with a range between 0 (worst) and 23 (best) points: scores ≤2 on the auditory, motor, and oromotor/verbal subscales, ≤1 on the visual subscale and = 0 on the communication subscale is consistent with the diagnosis of UWS. Higher scores (till 4 on the auditory, 5 on the visual and motor, 3 on the oromotor/verbal or 1 on the communication subscale) are consistent with the MCS diagnosis. More elevated scores in motor and/or communication subscales for two consecutive administrations of the scale indicate emergence from MCS.

### Experimental procedure

Data acquisition was performed in the morning, between 8:00 and 10:00 AM, in non-fasting state. First, all patients underwent a physical examination by an expert neurologist, during which a 12-lead standard ECG (mod. Delta-1 Plus, Cardioline, Italy) was executed, and axillary body temperature, blood O_2_ saturation (portable pulseoxymeter mod. Fingertip, Oscimed, Switzerland), systolic BP (SBP) and diastolic BP (DBP) (standard mercury sphygmomanometer) were measured. None of the subjects showed clinical sign of autonomic disease, like HR>120 bpm, SBP>160 mmHg, hypo/hyperthermia, sweating excess, cutaneous rushes.

Then, all patients were instrumented by a thoracic belt, connected to a piezo-resistive respiratory transducer (mod. TSD201, Biopack, Aero Camino Goleta, USA) to monitor rib cage movements. Arterial BP at the finger artery was recorded by applying a finger cuff around the middle finger of a hand (Finometer Pro, Finapres Medical System, the Nederlands). ECG was continuously monitored by a V_5_ lead. BP, ECG and ventilatory signals were digitized by the analog I/O system of the Finometer Pro device, at 200 Hz sampling frequency. Recordings were performed with the patients in recumbent position. Ten minutes after the start of the recording, a nociceptive stimulus was applied to the hand not instrumented by the Finometer cuff. The noxious stimulus consisted in a constant pressure produced by a load of 3.5 kg, applied for about 5 s on one fingernail bed by a validated Newton-meter (mod. Force Dial; Greenwich, USA). The recording continued for at least two minutes after the application of the stimulus.

During the delivery of the noxious stimulus an expert neuropsychologist administered the validated Nociception Coma Scale (NCS)[8]. NCS score ranges between 0 and 12 points and is based on the observation of behaviours as changes in facial expression and in mental status (grimace, cry, etc.), vocalizations or body movements in patients with consciousness disorders [10].

Data were stored in a protected database for offline analysis. Finger arterial BP was processed with the Beatscope software (Finapres Medical Systems B.V., Amsterdam, The Netherlands) that reconstructed the BP wave at the level of the brachial artery and calculated SBP, DBP, HR, stroke volume (SV) and total peripheral resistances (TPR) on a beat-by-beat base. The R-R interval (RRI) was derived beat by beat from the ECG.

#### Fast autonomic response to nociception

**Fig 1:**
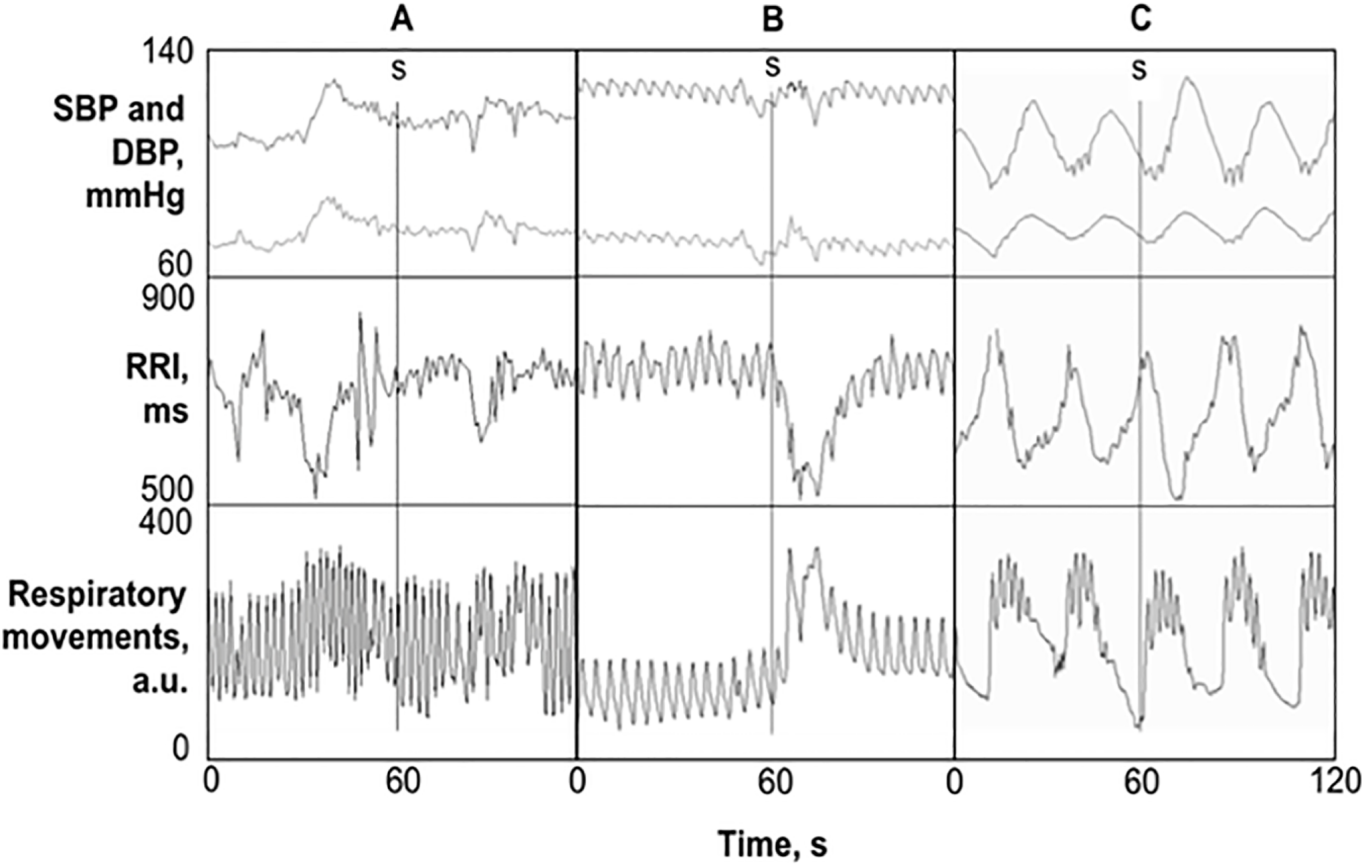
Examples of 2-minute data segments. Beat-by-beat series of SBP, DBP (upper panels), and RRI (central panels) and respiratory movements of the thorax (lower panels) in a patient classified as not-responder to the noxious stimulus (column A), as responder (column B), and as not-responder with periodic breathing (column C). The nociceptive stimulus, indicated by a vertical line and the letter “s”, was delivered in the middle of each time window (t = 60 s).

Two independent observers, both blinded to the consciousness status of the patient, visually inspected the recorded signals off-line to identify the presence of a fast autonomic response, synchronous with the delivery of the stimulus (see Fig 1). Patients’ response to nociception was classified as positive in case both observers recognized a clear decrease in RRI, or an increase in BP, or in amplitude or frequency of rib cage movements.

#### Short-term autonomic response

**Fig 2:**
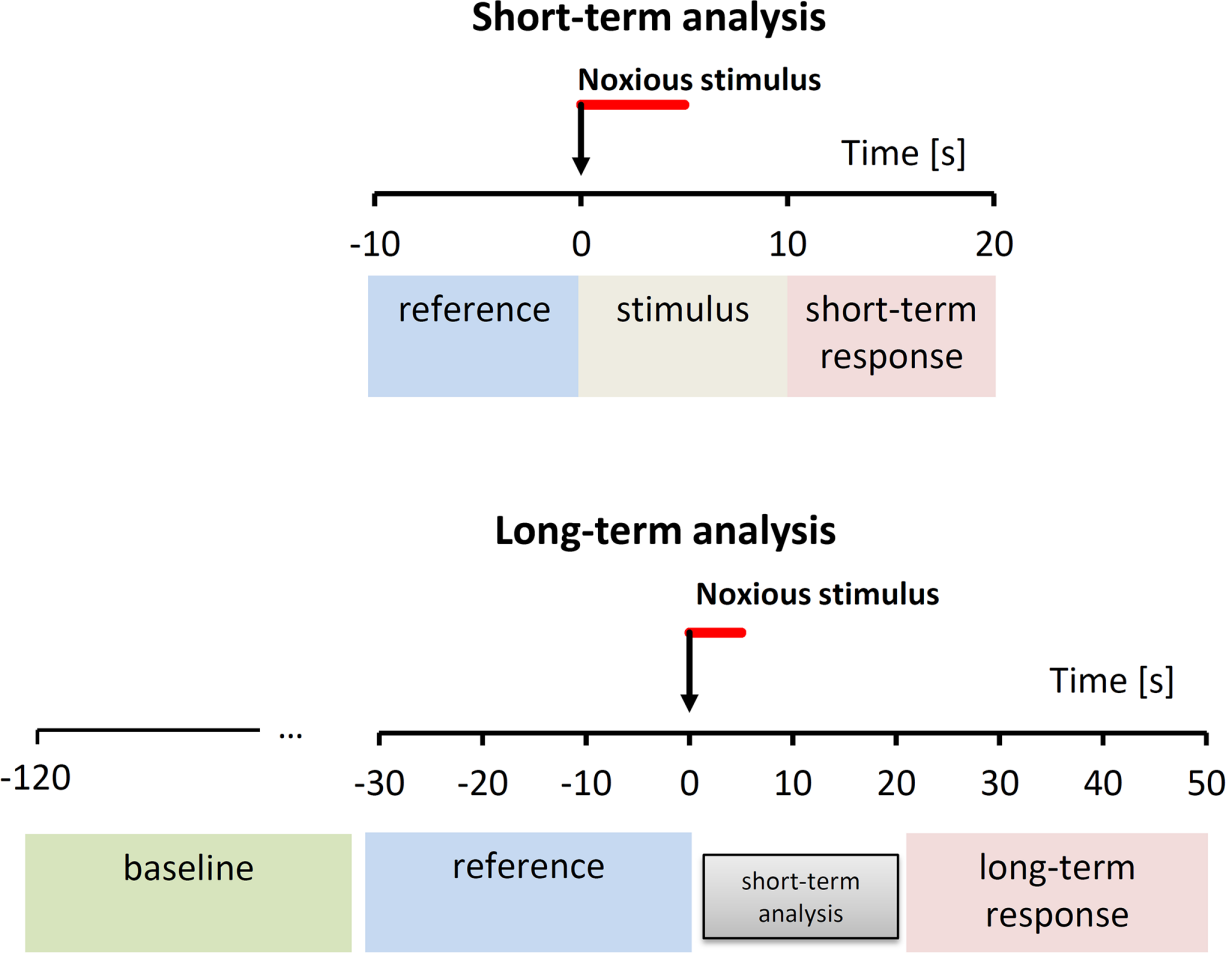
Reference and response periods for short-term and long-term analysis. Time series are split into consecutive blocks of 10 s; short- and long-term autonomic responses are quantified as increments from “reference” to “response” periods. The arrows indicate the application of the stimulus, with its 5-s duration highlighted by the red bar. References are the 10-s period (short-term) or 30-s period (long-term) preceding the stimulus; response periods have the same length of reference periods and start few seconds after the cessation of the stimulus (short-term response) or after the period selected for the short-term analysis (long-term response). Short-term analysis also quantifies changes during the “stimulus” condition; long-term analysis also consider a 90-s baseline period for evaluating if the operators interacting with the patient before the administration of the stimulus may have influenced the variables measured in the reference period.

Short-term changes induced by the noxious stimulus were assessed by comparing the average values of SBP, DBP, HR, SV and TPR time series calculated over three consecutive, non-overlapping data windows of 10-s (“reference”, “stimulus” and “short-term response”), with the second one (“stimulus”) starting synchronous with the delivery of the noxious pressure. Given the duration of 10 seconds, the “stimulus” data window completely covers the 5-s period of stimulus administration. Increments from the reference period to respectively the stimulus period and the short-term response period (Fig 2) were quantified for each beat-by-beat variable.

#### Long-term autonomic response

To assess the presence of a longer autonomic response to the nociception stimulus, and the possible presence of a “preparatory” phase before the stimulus, caused by the operators interacting with the patient, the profile of the nociception response was obtained around the stimulus administration. This was done by calculating the mean value of beat-by-beat parameters on a “baseline” subperiod defined in the time range between -2 minutes and -30 s before the application of the stimulus, and on other 8 consecutive sub-periods of 10-second length around the stimulus at *t* = 0 (time range: from -30 s to +50 s). To quantify the long-term autonomic response, the response period was defined between +20 s and +50 s after the stimulus, in this way following the short-term response period without overlapping, and the reference period was the data segment of equal length immediately preceding the stimulus. The long-term autonomic response was quantified as increment from the reference to the response period (Fig 2).

### Statistical analysis

UWS and MCS groups were compared by unpaired Student’s t test for cardiovascular and anthropometric variables; by Mann Whitney U test for NCS scores; and by the Fisher’s exact test for proportions.

To assess changes due to short-term autonomic response to the noxious stimulus, a mixed analysis of variance (ANOVA) with within-factor Time (3 repeated measures: “reference”, “stimulus” and “response”) and between-factor State (MCS or UWS) was performed on HR, SV, SBP, DBP and TPR, with Newman-Keuls post-hoc analysis.

To assess changes due to the long-term autonomic response to pain, mixed ANOVA was performed considering Time (9 repeated measures: “baseline”, -30, -20, -10, 0, +10, +20, +30 e +40) and State as factors, with Newman-Keuls post-hoc analysis. The hypothesis of Gaussian distribution was preliminary verified in baseline by the Kolmogorov-Smirnov test for each cardiovascular parameter (HR, SV, SBP, DBP and TPR).

The associations between NCS scores and short/long-term autonomic responses to the noxious stimulus were assessed by linear correlation. The significance level was set at p<0.05 and analyses were performed with Statistica 7.1 (StatSoft, Tulsa, OK, USA).

## Results

### Baseline data and NCS

Based on preliminary CRS-r scores, 6 patients were diagnosed in MCS and 14 in UWS. Table 1 reports anthropometric and clinical features of the two groups.

**Table 1:** Anthropometric and clinical data.

	UWS	MCS	*P* value
**N**	14	6	
**CRS-r, score**	5±1	11 ±6	
**Gender, M/F**	8/6	2/4	0.63
**Age, years**	54±15	56±13	0.76
**Weight, kg**	58±8	57±6	0.71
**Height, cm**	170±10	162±9	0.14
**BMI, kg m**^**-2**^	20.3±3.0	21.9±4.0	0.40
**SBP, mmHg**	112±6	110±1	0.31
**DBP, mmHg**	68±5	65±9	0.58
**HR, bpm**	80±5	77±5	0.52
**Body temperature, °C**	36.4±0.3	36.4±0.3	0.96
**Blood O**_**2**_ **saturation, %**	96±1	96±1	0.97

Anthropometric and clinical data (mean±SD) of UWS and MCS patients. BMI = body mass index; see text for other abbreviations.

Recordings in 2 UWS and 1 MCS patients were discarded from subsequent analysis because muscle hypertonia and uncontrolled movements did not allow applying properly the noxious stimulus or obtaining finger BP recordings of sufficiently good quality for the analysis.

**Fig 3:**
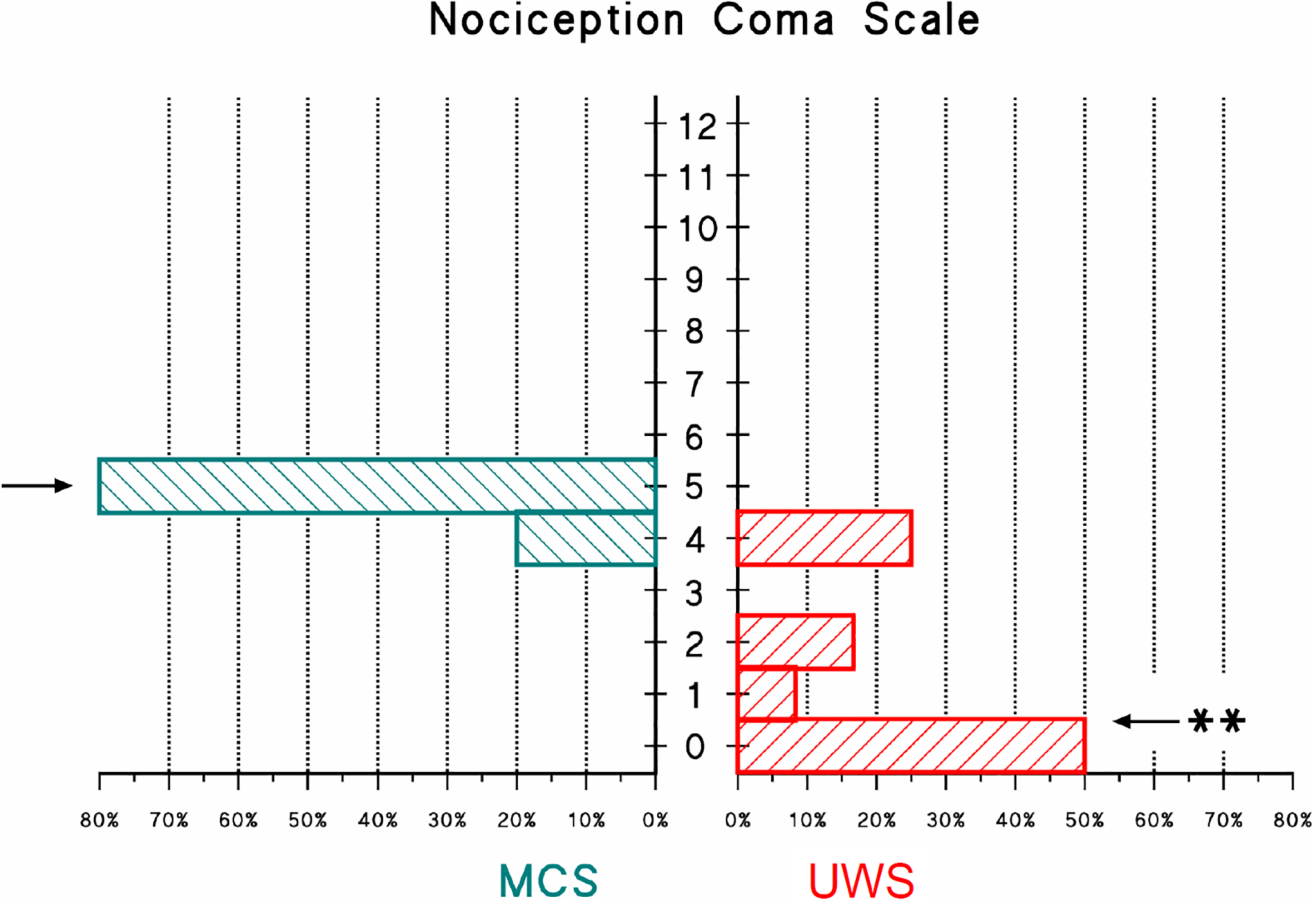
Frequency distribution of NCS scores. Frequency distribution is shown for the MCS (left histogram) and UWS (right histogram) groups; the arrows show the median values of each distribution; the “**” indicates a statistically significant difference between distributions at p<0.01 (Mann Whitney U test).

The histogram of NCS scores evaluated in the remaining 17 patients revealed a statistically different distribution of the behavioral responses to pain between groups (Fig 3). Individual NCS scores are reported in S1 Table.

### Fast autonomic response

About half of the patients (47%) elicited a fast autonomic reaction to the pain stimulus and were classified as “responders”. Percentage of responders was slightly higher in the MCS (60.0 ± 21.9%, percentage ± SD) than in the UWS group (41.7 ± 14.2%) but the difference was not significant (p = 0.62). Visual inspection of ventilatory movements showed the occurrence of periodic breathing (see Fig 1) in 5 out of these 17 patients. Interestingly, it was not possible to observe a fast autonomic response to pain in any of the patients with periodic breathing (4 in UWS and 1 in MCS). However, even removing these patients from the analysis, the difference between UWS and MCS groups in the percentage of responders was not significant.

Since periodic breathing induces synchronous oscillations in RRI, SBP and DBP that alter importantly the dynamics of cardiovascular time series, patients with periodic breathing were excluded from the following analyses. The reasons for the exclusion are clear looking at Fig 1: the decrease in RRI that follows the noxious stimulus in the responder patient has magnitude and duration similar to the rhythmic RRI oscillations induced by periodic breathing, which are therefore indistinguishable from a possible response to pain. Similar changes occur for DBP. As to SBP, oscillations induced by periodic breathing are even much larger than the SBP change following the stimulus in the responder patient, so that that they could totally obscure an eventual autonomic responses to pain. Therefore, short-term and long-term autonomic response to pain were assessed on the remaining 12 patients, 4 in MCS and 8 in UWS.

### Short-term autonomic response

The analysis of the autonomic response within 20 s from the application of the stimulus identified as statistically significant the factor time for TPR, SBP and DBP. However, the interaction between time and state was significant for TPR only (Table 2).

**Table 2:** Factor significance for autonomic response.

	Short-Term Response			Long-Term Response		
	Time	State	Interaction	Time	State	Interaction
**HR**	0.14	0.63	0.15	<0.05	0.58	<0.001
**SBP**	<0.01	0.31	0.54	0.13	0.47	0.62
**DBP**	<0.01	0.30	0.28	<0.05	0.39	0.70
**SV**	0.20	0.85	0.17	0.99	0.93	0.87
**TPR**	<0.01	0.85	<0.05	0.56	0.73	0.38

Factors significance (ANOVA) for short-term and long-term autonomic nociception response.

**Fig 4:**
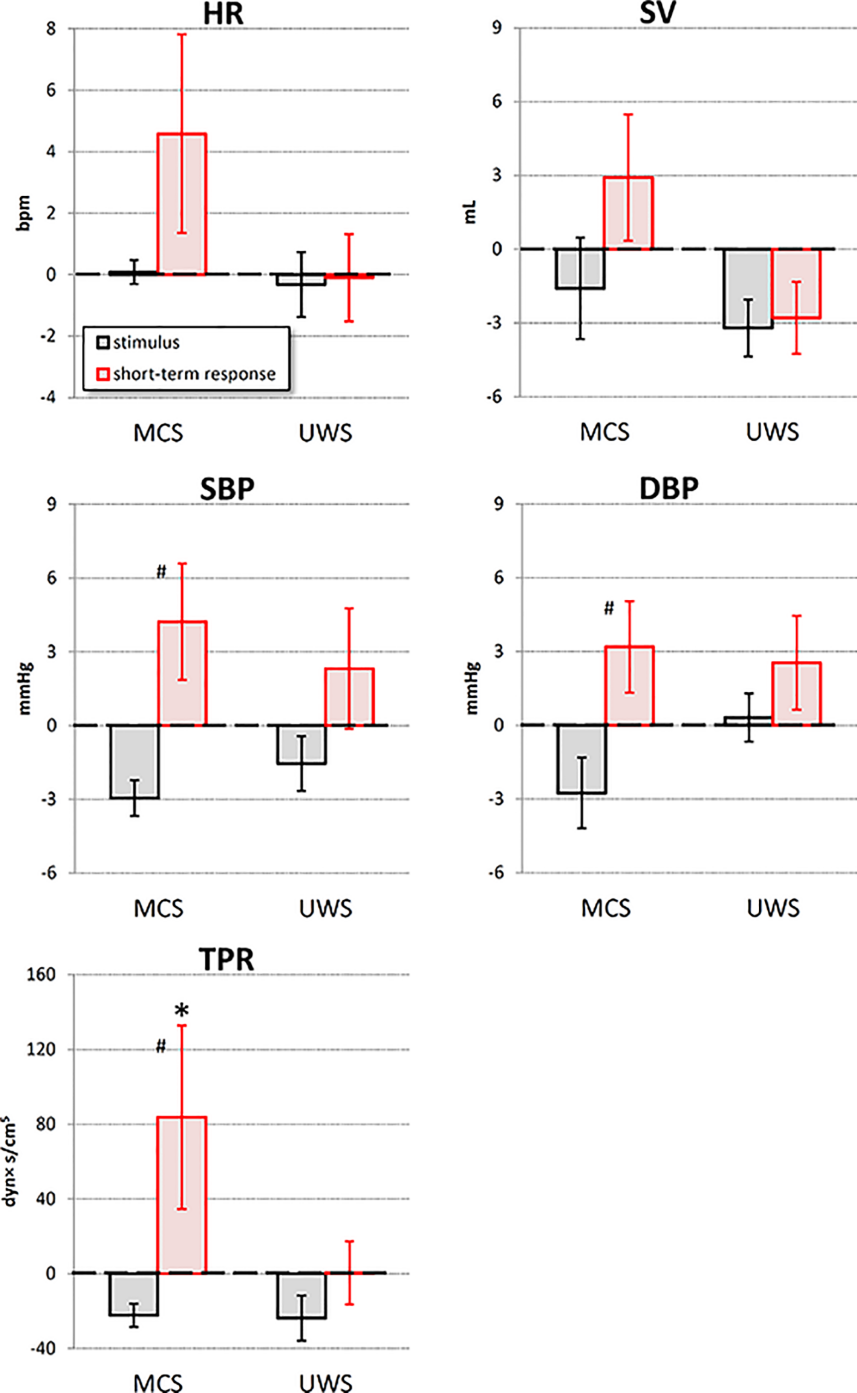
Short–term analysis of the noxious stimulus in MCS and UWS patients. Grey bars: increments from “reference” to “stimulus” condition; red bars: increments from “reference” to “response” condition: values as mean ± sem; the * indicates increments significantly different from 0, the # indicates significant differences between “stimulus” and “response” increments (p<0.05).

Post-hoc analysis showed that the noxious stimulus increased TPR from the reference to the short-term response condition in the MCS group only (Fig 4). Moreover, in the MCS group only TPR was higher in the short-term response than in the stimulus condition, a trend appearing to also characterize SBP and DBP of the MCS group. Individual increments from reference to stimulus condition and from reference to short-term response are reported in S2 Table.

### Long-term autonomic response

**Fig 5:**
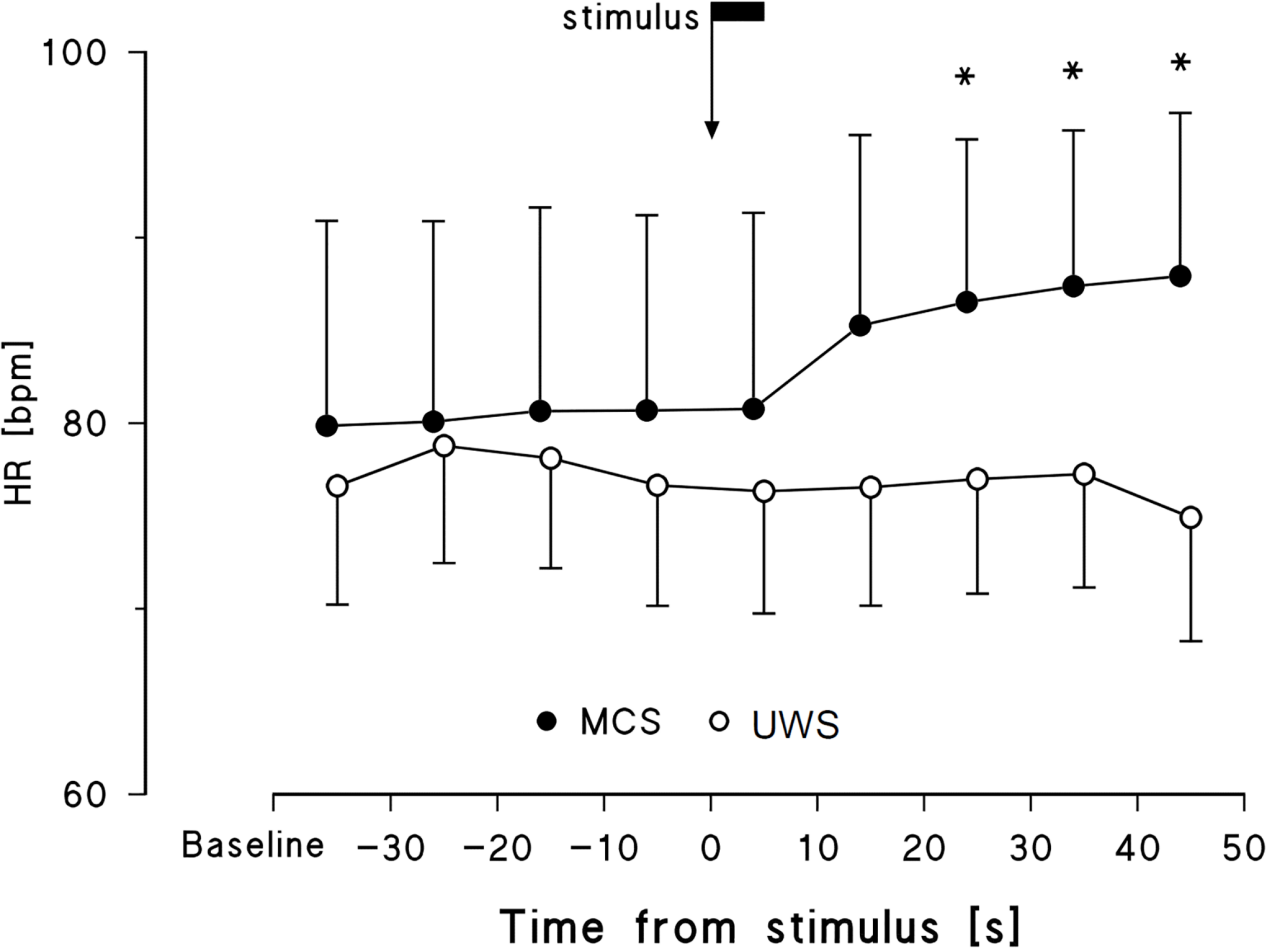
HR profile around the noxious stimulus in MCS and UWS patients. Data are mean and sem. The arrow pointing at *t* = 0 indicates the instant of stimulus application; the flag on the arrow indicates the duration of the stimulus (5 s); the * mark differences vs. baseline significant at p<0.05.

The analysis of the long-term response (i.e., between 20 and 50 s from the application of the stimulus) identified the factor *Time* and the interaction between *Time* and *Status* as significant for HR only (Table 2), suggesting that the HR long-term response to the noxious stimulus may differentiate between MCS and UWS. The HR profile showed a persistent increase following the noxious stimulus in the MCS group only, particularly evident 20 s after the administration of the stimulus, without any indication for a preparatory autonomic response (Fig 5). Individual increments from reference to stimulus condition and from reference to long-term response are reported in S3 Table.

**Fig 6:**
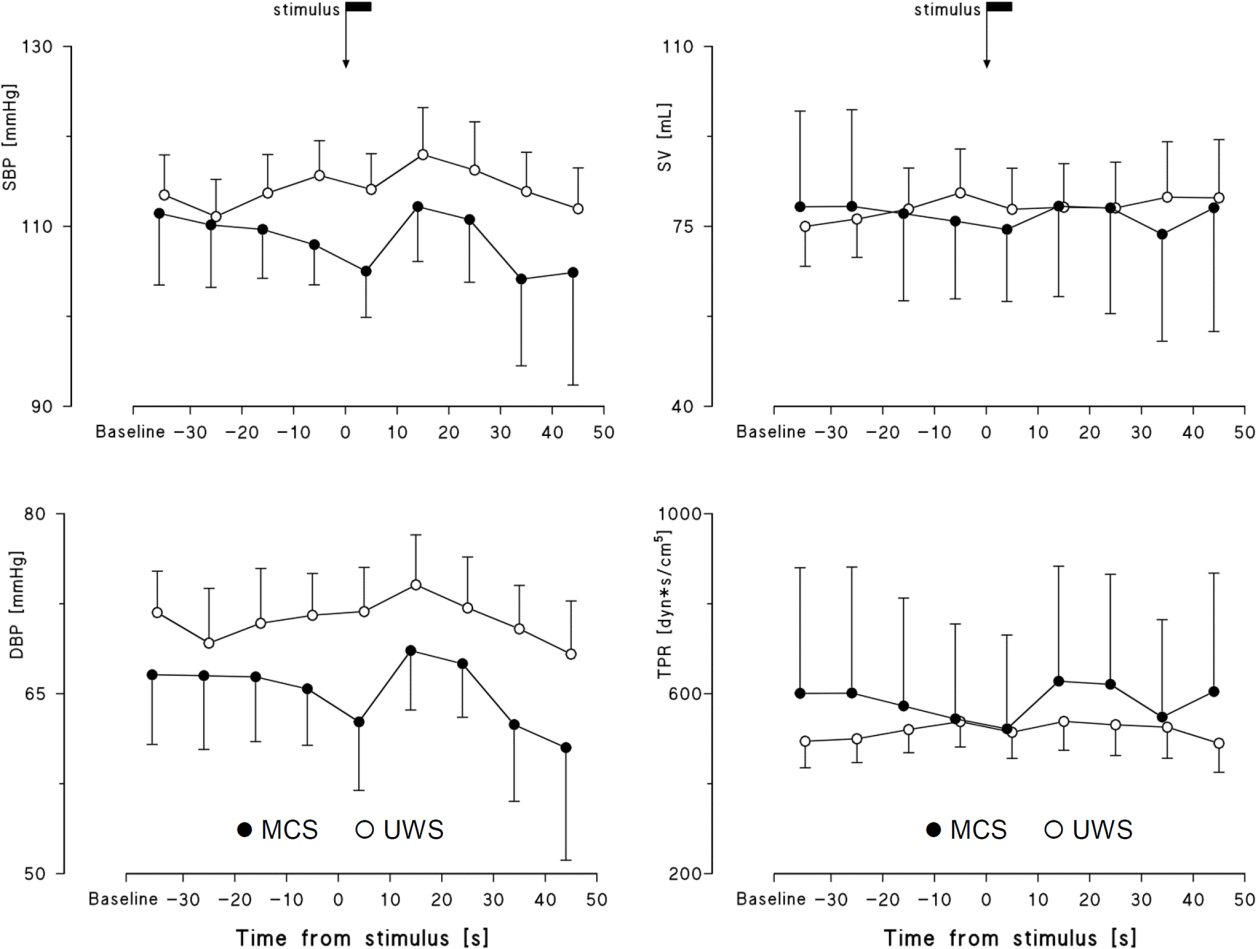
SBP, DBP, SV and TPR profiles around the noxious stimulus. Data as mean and sem; the arrow indicates the stimulus application. No differences vs. baseline were significant at p<0.05.

The time courses of SBP, DBP, SV and TPR did not evidence any trend that might differentiate the MCS and UWS groups (Fig 6, Table 2).

Table 3 synthesizes the short-term and long-term responses to the noxious stimulus as percentage of patients with an increment from the reference condition of at least 10 dyn×s/cm^5^ for TPR (as short-term response), or of at least 1 bpm for HR (as long-term response), separately for MCS and UWS patients.

**Table 3:** Percentage of patients with non-negligible positive response to the noxious stimulus.

	Short-Term Response ΔTPR> 10 dyn×s/cm^5^	Long-Term Response ΔHR>1 bpm
**MCS**	75%	100%
**UWS**	25%	12.5%

### Relationship between NCS and short/long-term autonomic responses

**Fig 7:**
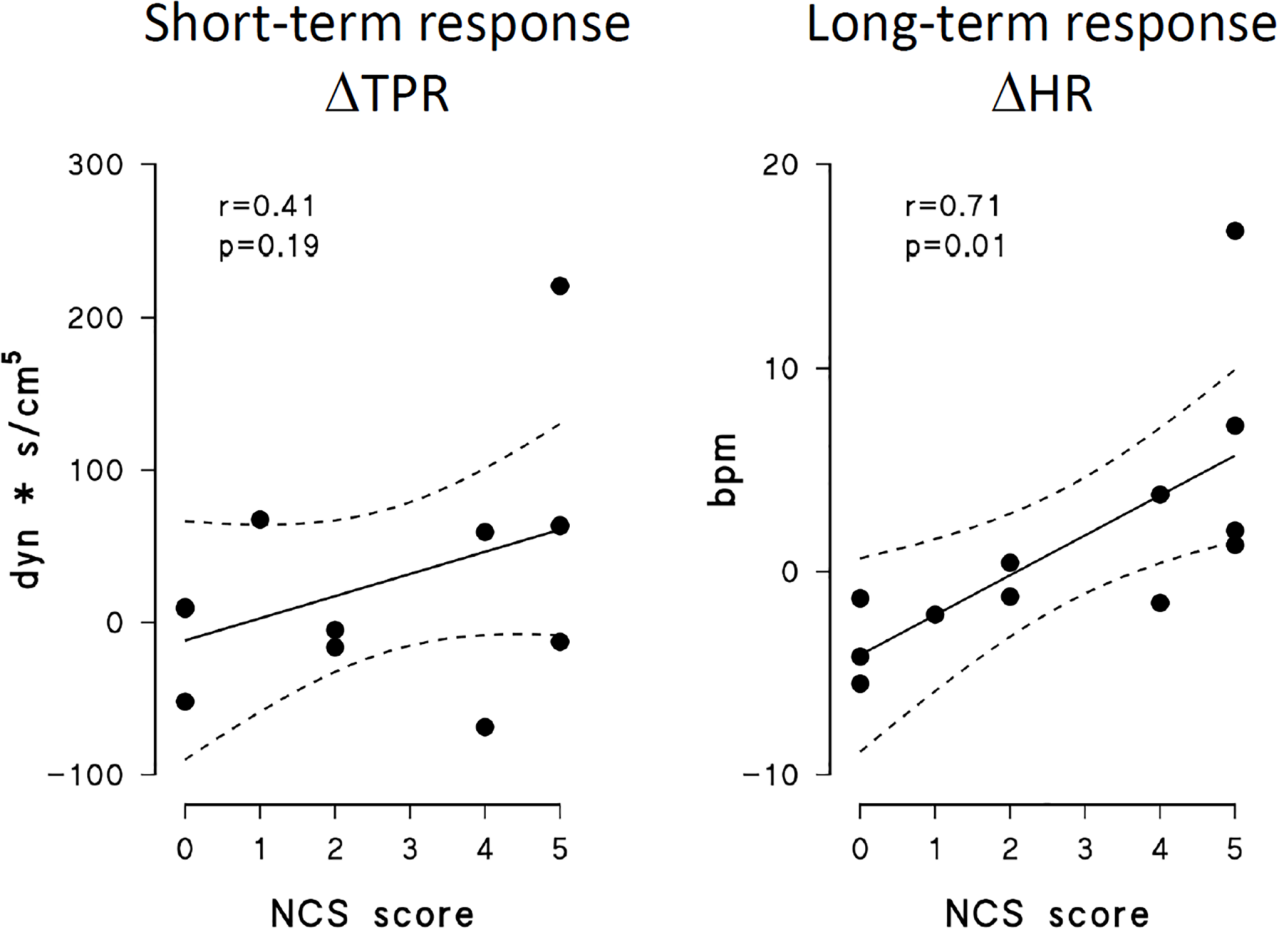
Correlation between NCS score and autonomic indices of short-term and long–term response to nociception stimulus. Left: short-term response in TPR; right: long-term response in HR.

Correlations between NCS scores and autonomic responses to pain are shown only for the autonomic variables where a significant interaction between time and state (Table 2) indicates a different response in MCS and UWS patients, i.e. for short-term ΔTPR (increase in TPR from the reference to the short-term response condition) and long-term ΔHR (increase in HR from reference to long-term response condition). The NCS score was significantly correlated with ΔHR but not with ΔTPR (Fig 7).

## Discussion

The main finding of this study is that the autonomic cardiovascular response to a nociceptive stimulus appears characterized by a short-term response (i.e., within 20 s after the start of the stimulus) and a long-term response (i.e., between 20 s and 50 s after the stimulus), both of them somehow differently activated by pain in MCS and UWS patients.

In our MCS patients, the short-term autonomic response parallels the typical cardiovascular response to acute pain, since we found a significant increase in BP and in TPR in the short-term response vs. the stimulus condition, and a tendency to increase for HR and SV (Fig 4). In fact, classic studies on nociception in humans demonstrated that a painful stimulus (cold pressor test) activates a sympathetic response, quantified as an increase in firing rate of peroneal nerve fibres, with increasing levels of circulating catecholamines [11]. This leads to an increase in HR and BP, and to the activation of peripheral vascular resistances [12]. Indeed, it has been demonstrated that painful events activate central noradrenergic circuits, and that these circuits play a role in the autonomic and central arousal associated with pain [13]. Our results in MCS patients are therefore in agreement with previous findings on the physiological reactions to pain. Interestingly, we found a significant interaction between the time of the response to the stimulus and the status of consciousness for TPR, and the UWS group did not show signs of a possible TPR increase in the short-term response condition vs. the stimulus or the reference condition. Moreover, as far as the short-term response is considered, also HR and SV did not show any sign of increase in the UWS group. These data suggest the involvement of higher cortical centers in the short-term autonomic response to pain, and an impairment of these higher centers responsible for their reduced or missing response in UWS patients.

However, the finding that the percentage of patients showing a fast response to the nociceptive stimulus does not differ significantly between MCS and UWS groups would suggest that the lower neural centers (likely those responsible for the fast response) preserve their functions similarly in the two groups of brain-injured patients. These results are in agreement with those of previous studies reporting that the autonomic changes in HR, BP and respiratory movements were not reliable indicators of response to nociception in individuals with severe brain damage [5].

Considering the response over a longer time scale, i.e., ten of seconds from the stimulus application, the picture substantially changes and we found a significant interaction for HR only. In fact, the HR level increased significantly about half a minute after the application of the painful stimulus, and then remained higher than baseline for tens of seconds, but in the MCS group only. The substantial lack of a long-term HR response in UWS patients probably reflects an extension of brain damage at higher levels compared to MCS patients. Indeed, magnetic resonance imaging studies showed that the central response to pain requires the integrity of different brain areas and of their neuronal connections, besides the preserved function of peripheral sensory organs. The transport of the nociceptive information to the higher levels of the central nervous system occurs through multiple and parallel upward projections that lead the signal from the spinal cord to prosencephalon, mesencephalon, and cortex [14]. The persistent increase of HR in MCS patients may be due to the preservation of the neural central pathways which vehicle the painful stimulus, and its observation in patients with brain damage may therefore suggest a positive clinical prognostic value. This is also in accordance with a differential temporal analysis of HR following nociception, demonstrating that the HR response to a painful stimulus reflects both 1) an initial brief “nocifensive reflex” lasting only few seconds and induced by the sensory component and, subsequently, 2) a longer (about 20 s) response probably related to affective and/or cognitive evaluation [15]. A prolonged HR activation following nociception may therefore represent another sign of consciousness preserved in MCS but not in UWS patients.

A second explanation for the persistent increase in HR is that MCS patients (differently from UWS individuals) may develop an “alert” state in response to pain. Although indirectly, this may reveal a different sensitivity to nociception and therefore a different residual functionality of the central nervous system function in MCS and UWS patients.

Finally, a third mechanism may have contributed to the long-term HR increase in MCS patients: a persistent increase in circulating catecholamines. Some studies demonstrated that catecholamines may increase excitability or sensitize nociceptors [16], thereby contributing to amplification and prolongation of pain, but it is unclear whether a single painful stimulus may cause a persistent increase in circulating adrenaline. Harden et al., for example, fails to demonstrate an increased release of cathecolamines by the adrenal medulla in conditions of sympathetically maintained pain [17].

Our MCS patients also showed a more pronounced “behavioural” response to the pain stimulus, as quantified by the NCS score, compared to the UWS group. This finding supports the hypothesis that MCS patients have a less compromised capacity to perceive pain compared with UWS patients. This aspect of pain response in MCS state deserves therefore further neuro-hormonal and behavioural investigations.

Interestingly, we found a significant correlation between the long-term increase in HR after the painful stimulus and the score of the behavioural response to the painful stimulus as provided by NCS. From a physiological viewpoint, the correlation suggests that the autonomic long-term response may involve the same complex brain processing centers devoted to "behavioral" responses to pain. This finding suggests that the HR long-term response could become the basis of an "automated" test for monitoring the patient’s consciousness status, simple to implement and relatively robust in terms of signal acquisition and processing. In fact, HR can be easily recorded, even on a beat-to-beat basis, for long periods of time, and currently available miniaturized devices can be safely used to this purpose. A test based on the HR response to pain might potentially provide information on the consciousness status similar to that scored by NCS, but in a less subjective way. Such a test could complement the NCS evaluation, providing a quantitative, long-term and operator-independent monitoring, without involving experienced testing staff.

On the other hand, the short-term increase in TPR was not significantly correlated with the NCS score. In this case, the lack of correlation suggests the involvement of centers different from those producing a behavioral response, as those activated by vascular reflexes. This suggest the possibility that the information provided by the short-term vascular response to pain is independent from the information provided by NCS, and that a test based on TPR changes may effectively integrate the NCS score. Therefore, it is conceivable that a test integrating the NCS score with the short-term vascular autonomic response to pain may effectively improve the patients’ assessment.

Such a test, based on the administration of a noxious stimulation, may have ethical implications that rise an important cost-benefit issue. It should be considered, however, that it may potentially provide relevant information in the face of a minimum painful stimulus. Furthermore, if its scientific validity will be confirmed by further studies, it could be used only occasionally in clinical practice, for instance to check the initial state of consciousness, being repeated only if the clinical conditions of the patient change.

Finally, some limitations should be considered. First, the autonomic response to a pain stimulus could be altered by the use of β-blockers that may blunt the adrenergic response to nociception. Actually, comatose patients, especially those suffering from traumatic injuries, are often treated with β-blocking agents, which reduce mortality caused by catecholamine surges often occurring in such patients [18]. However, in our study the autonomic cardiorespiratory response to pain was positive in 36% of the patients taking β-blocking agents and in 67% of the patients not treated with anti-adrenergic drugs, independently from their consciousness state. This finding, even if not excluding that β-blockers may have altered the response to the noxious stimulus, would nevertheless suggest that they did not prevent the identification of the autonomic activation.

Second, the beat-by-beat BP response to the nociceptive stimuli could not be recorded with the finger cuff in patients in which a marked hypertonia of the upper limb muscles resulted in extreme flexion of the hand fingers. However, this is not a major limitation because our study points out that information for discriminating between MCS and UWS is also associated with the long-term response of HR.

Third, periodic breathing induces HR changes that make difficult or even impossible to observe a reliable cardiorespiratory reaction to pain, suggesting that a test based on the autonomic response to pain cannot provide useful information for discriminating between MCS and UWS in this fraction of patients.

## Supporting information

S1 Table Individual scores of the Nociception Coma Scale. (DOCX)

S2 Table Short-term analysis: Individual increments from “reference” to “stimulus” and to “response” conditions. Individual increments of the parameters HR, SBP, DBP, SV and TPR from “reference” to “stimulus” and to “response” conditions in the short-term analysis.
(DOCX)

S3 Table Long-term analysis: Individual values in “reference” and “response” conditions. Individual increments of the parameters HR, SBP, DBP, SV and TPR from “reference” to “stimulus” and to “response” conditions in the long-term analysis.
(DOCX)
