# Supplementary material for: Cardio-respiratory autonomic responses to nociceptive stimuli in patients with disorders of consciousness
Source: PLoS One. 2018 Sep 12;13(9):e0201921. doi: 10.1371/journal.pone.0201921 (PMC6135369; doi:10.1371/journal.pone.0201921)
Supplement: S1 Table — (DOCX) [file pone.0201921.s001.docx]

**S1 Table. Individual Scores of the Nociception Coma Scale**

| **ID** | **Case** | **Periodic Breathing** | **NCS Score** |
| --- | --- | --- | --- |
| 1 | MCS | no | 5 |
| 2 | MCS | no | 5 |
| 3 | MCS | no | 5 |
| 4 | MCS | no | 5 |
| 5 | MCS | yes | 4 |
| 6 | UWS | no | 1 |
| 7 | UWS | no | 2 |
| 8 | UWS | no | 4 |
| 9 | UWS | no | 0 |
| 10 | UWS | no | 0 |
| 11 | UWS | no | 2 |
| 12 | UWS | no | 4 |
| 13 | UWS | no | 0 |
| 14 | UWS | yes | 0 |
| 15 | UWS | yes | 4 |
| 16 | UWS | yes | 0 |
| 17 | UWS | yes | 0 |
