# Supplementary material for: Cardio-respiratory autonomic responses to nociceptive stimuli in patients with disorders of consciousness
Source: PLoS One. 2018 Sep 12;13(9):e0201921. doi: 10.1371/journal.pone.0201921 (PMC6135369; doi:10.1371/journal.pone.0201921)
Supplement: S2 Table — Individual increments of the parameters HR, SBP, DBP, SV and TPR from “reference” to “stimulus” and to “response” conditions in the short-term analysis. (DOCX) [file pone.0201921.s002.docx]

**S2 Table. Short-Term Analysis: Individual Increments from “reference” to “stimulus” and to “response” conditions.** Individual increments of the parameters HR, SBP, DBP, SV and TPR from “reference” to “stimulus” and to “response” conditions in the short-term analysis.

|  |  | HR (bpm) | | SBP (mmHg) | | DBP (mmHg) | | SV (mL) | | TPR (dyn*s/cm^5^) | |
| --- | --- | --- | --- | --- | --- | --- | --- | --- | --- | --- | --- |
| ID | Case | Stimulus | Response | Stimulus | Response | Stimulus | Response | Stimulus | Response | Stimulus | Response |
| 1 | MCS | 0.90 | 1.00 | -3.55 | -2.50 | -3.20 | -1.90 | -1.04 | -0.82 | -18.84 | -12.78 |
| 2 | MCS | -0.90 | 14.10 | -3.40 | 4.40 | -6.45 | 6.40 | 3.68 | -2.01 | -7.00 | 63.16 |
| 3 | MCS | -0.11 | 3.13 | -0.80 | 7.90 | 0.46 | 5.44 | -2.83 | 8.62 | -27.19 | 220.76 |
| 4 | MCS | 0.45 | 0.10 | -4.05 | 7.10 | -1.85 | 2.80 | -6.21 | 5.86 | -36.08 | 63.67 |
| 6 | UWS | 6.35 | 5.15 | -0.60 | 6.10 | 3.55 | 7.00 | -9.48 | -3.08 | 5.38 | 67.42 |
| 7 | UWS | -0.21 | 0.89 | -0.21 | 2.92 | -0.47 | 4.29 | 0.62 | -3.98 | -1.93 | -5.02 |
| 8 | UWS | -1.95 | 4.85 | 2.75 | 16.65 | 3.15 | 11.05 | -3.33 | -10.73 | -26.57 | 59.23 |
| 9 | UWS | 0.79 | 0.07 | 1.14 | 2.86 | 1.93 | 3.07 | -2.40 | -2.54 | 8.88 | 8.89 |
| 10 | UWS | -1.00 | -0.42 | -2.75 | -1.04 | -1.08 | -2.46 | -2.18 | 4.47 | -25.81 | 9.94 |
| 11 | UWS | -0.75 | -0.60 | -0.85 | -2.35 | -1.45 | -2.15 | 0.55 | -1.30 | -3.97 | -16.40 |
| 12 | UWS | -3.50 | -6.75 | -5.50 | -0.25 | 1.63 | 4.50 | -4.25 | -1.86 | -87.97 | -68.71 |
| 13 | UWS | -2.30 | -4.05 | -6.35 | -6.35 | -4.75 | -5.00 | -5.19 | -3.38 | -58.12 | -52.02 |
