# Supplementary material for: Cardio-respiratory autonomic responses to nociceptive stimuli in patients with disorders of consciousness
Source: PLoS One. 2018 Sep 12;13(9):e0201921. doi: 10.1371/journal.pone.0201921 (PMC6135369; doi:10.1371/journal.pone.0201921)
Supplement: S3 Table — Individual increments of the parameters HR, SBP, DBP, SV and TPR from “reference” to “stimulus” and to “response” conditions in the long-term analysis. (DOCX) [file pone.0201921.s003.docx]

**S3 Table. Long-Term Analysis: Individual values in “reference” and “response” conditions.** Individual increments of the parameters HR, SBP, DBP, SV and TPR from “reference” to “stimulus” and to “response” conditions in the long-term analysis.

|  |  | HR (bpm) | | SBP (mmHg) | | DBP (mmHg) | | SV (mL) | | TPR (dyn*s/cm^5^) | |
| --- | --- | --- | --- | --- | --- | --- | --- | --- | --- | --- | --- |
| ID | Case | Reference | Response | Reference | Response | Reference | Response | Reference | Response | Reference | Response |
| 1 | MCS | 85.72 | 87.73 | 105.69 | 102.49 | 70.25 | 67.82 | 53.92 | 53.50 | 385 | 376 |
| 2 | MCS | 66.42 | 83.13 | 100.07 | 81.23 | 58.22 | 47.05 | 51.81 | 28.00 | 251 | 144 |
| 3 | MCS | 108.61 | 109.91 | 125.45 | 123.71 | 79.43 | 78.58 | 124.42 | 121.11 | 1298 | 1264 |
| 4 | MCS | 61.15 | 68.33 | 105.83 | 118.92 | 56.49 | 60.43 | 79.66 | 104.90 | 357 | 580 |
| 5 | UWS | 72.40 | 70.28 | 108.20 | 107.39 | 60.75 | 58.17 | 101.33 | 105.80 | 567 | 557 |
| 6 | UWS | 115.54 | 115.97 | 99.72 | 96.94 | 59.61 | 60.61 | 63.04 | 53.88 | 537 | 459 |
| 7 | UWS | 56.58 | 60.37 | 132.11 | 139.01 | 85.89 | 84.40 | 103.74 | 113.03 | 613 | 720 |
| 8 | UWS | 82.76 | 78.57 | 121.19 | 123.63 | 83.77 | 86.17 | 89.43 | 88.27 | 711 | 686 |
| 9 | UWS | 79.39 | 78.07 | 104.62 | 113.16 | 58.01 | 62.55 | 100.31 | 115.23 | 605 | 721 |
| 10 | UWS | 69.15 | 67.92 | 103.97 | 100.97 | 62.76 | 60.49 | 47.39 | 44.30 | 254 | 224 |
| 11 | UWS | 81.13 | 79.58 | 119.55 | 116.13 | 73.87 | 73.66 | 65.17 | 62.85 | 490 | 443 |
| 12 | UWS | 65.85 | 60.33 | 118.50 | 115.00 | 79.64 | 76.19 | 59.79 | 56.15 | 377 | 312 |
